# Variations in susceptibility to common insecticides and resistance mechanisms among morphologically identified sibling species of the malaria vector *Anopheles subpictus *in Sri Lanka

**DOI:** 10.1186/1756-3305-5-34

**Published:** 2012-02-10

**Authors:** Sinnathamby N Surendran, Pavilupillai J Jude, Thilini C Weerarathne, SHP Parakrama Karunaratne, Ranjan Ramasamy

**Affiliations:** 1 Department of Zoology, Faculty of Science, University of Jaffna, Jaffna, Sri Lanka; 2 Department of Zoology, Faculty of Science, University of Peradeniya, Peradeniya, Sri Lanka; 3 Institute of Health Sciences, Universiti Brunei Darussalam, Gadong, Brunei Darussalam

**Keywords:** *Anopheles subpictus s.l.*, insecticide resistance, resistance mechanism, sibling species, Sri Lanka

## Abstract

**Background:**

*Anopheles subpictus s.l*., an important malaria vector in Sri Lanka, is a complex of four morphologically identified sibling species A-D. Species A-D reportedly differ in bio-ecological traits that are important for vector control. We investigated possible variations that had not been reported previously, in the susceptibility to common insecticides and resistance mechanisms among the *An. subpictus *sibling species.

**Methods:**

Adult *An. subpictus *were collected from localities in four administrative districts in the dry zone of Sri Lanka. Single female isoprogeny lines were established and sibling species status determined according to reported egg morphology. World Health Organization's standard protocols were used for insecticide bioassays and biochemical assays to determine insecticide susceptibility and resistance mechanisms. Susceptibility of mosquitoes was tested against DDT (5%), malathion (4%), deltamethrin (0.05%) and λ-cyhalothrin (0.05%). Biochemical basis for resistance was determined through assaying for esterase, glutathione-S-transferase and monooxygenase activities and the insensitivity of acetycholinesterase (AChE) to propoxur inhibition.

**Results:**

All sibling species were highly resistant to DDT. However there were significant differences among the sibling species in their susceptibility to the other tested insecticides. Few species A could be collected for testing, and where testing was possible, species A tended to behave more similarly to species C and D than to B. Species B was more susceptible to all the tested insecticides than the other sibling species. This difference may be attributed to the predominance of species B in coastal areas where selection pressure due to indoor residual spraying of insecticides (IRS) was lower. However there were significant differences between the more inland species C and D mainly towards pyrethroids. Higher GST activities in species C and D might have contributed to their greater DDT resistance than species B. Malathion resistance in both species C and D may be caused by elevated GST activity and an altered insensitive target site in AChE. In addition, a carboxylesterase based malathion resistance mechanisms was also detected in species C and D. Elevated esterase levels in species C and D might have contributed to the low levels of pyrethroid resistance. However an absence of elevated activity of monooxygenases in species B, C and D indicates that monooxygenases are unlikely to be the cause of this partial resistance to pyrethroids.

**Conclusions:**

The differences in insecticide susceptibility and insecticide resistance mechanism shown by *An. subpictus *sibling species are important considerations for developing the malaria control and eradication program in Sri Lanka. Similar studies on species complexes of other anopheline vectors of malaria are necessary for effective malaria control worldwide. The differential susceptibility findings are also consistent with most, if not all, morphologically identified *An. subpictus *species B in Sri Lanka belonging to the *An. sundaicus *complex. There is a need therefore to develop molecular techniques that can be used to differentiate morphologically similar anopheline species in field conditions for more effective vector control.

## Background

*Anopheles culicifacies *species E is the primary vector of falciparum and vivax malaria in Sri Lanka [1,2] but *An. subpictus s.l*. is the major vector in the Jaffna district [3], and an important secondary vector elsewhere in Sri Lanka [2-5], India and Southeast Asia [6-9]. The taxon *An. subpictus *is reported to be a complex of four sibling species, viz. A, B, C and D in India that can be differentiated through morphological and chromosomal characteristics [9,10]. Members of the Subpictus Complex also show differences in bio-ecological traits such as vectorial capacity, preimaginal development habitats, feeding preferences and salinity tolerance, that constitute important entomological properties for developing vector control programmes [2,6,8,11]. Studies on the numbers of egg ridges and X-chromosome inversions that characterize the Indian *An. subpictus *sibling species suggest that all four sibling species are present in Sri Lanka [12,13]. However DNA sequences of ribosomal RNA genes have recently shown that most, if not all, *An. subpictus *species B identified morphologically in East Sri Lanka through the characteristic number of egg ridges attributed to the Indian species B are in fact members of the *An. sundaicus *complex [14]. These are therefore referred to as *An. subpictus *B/*An. sundaicus s.l*. in this article until their taxonomic status is finally determined.

Indoor residual spraying of insecticides (IRS) has been the major vector control measure undertaken by the Anti Malaria Campaign (AMC) of the Ministry of Health, Sri Lanka since the middle of the 20^th ^century. Dichloro-diphenyl-trichloroethane (DDT), a synthetic organochloride, was used until mid 1970s, when due to the development of DDT resistance it was replaced by the synthetic organophosphate (OP) malathion. The use of malathion in IRS was discontinued in 1993 in most parts of the country as a result of the development of resistance, and replaced with λ-cyhalothrin, a synthetic pyrethroid. At present insecticides such as fenitrothion, λ-cyhalothrin, cyfluthrin, deltamethrin and etofenprox are used in different districts on rotational basis to delay the development of resistance in mosquitoes. In addition to IRS, long lasting insecticide- treated nets are distributed by the AMC as a supplementary malaria control measure [15].

Mosquitoes develop resistance by elevating enzyme activity to detoxify insecticides, sequestering insecticides away from their target sites or by mutating the target site to negate insecticide binding and effector function [16-18]. Monitoring resistance development and establishing its underlying mechanisms in vector populations are therefore important for maintaining effective vector control. Studies have previously been carried out to establish the resistance status of *An. culicifacies s.l*. and *An. subpictus s.l*. against commonly used insecticides such as DDT, malathion, deltamethrin, λ-cyhalothrin etc. in Sri Lanka [19-21]. However, the two taxa exist as species complexes in Sri Lanka [2] and therefore the spatio-temporal variations in resistance to different insecticides that were observed [19,21] may have partly been caused by changing prevalence of the different sibling species in the island. A previous study on the differential susceptibility of the two members of the *An. culicifacies *complex present in Sri Lanka showed that vector species E was more resistant to malathion than the non-vector species B [22]. Another study that investigated insecticide resistance mechanisms among *An. subpictus s.l*. populations demonstrated a degree of heterogeneity that was attributed to the presence of sibling species [19]. However there is presently no information on differential resistance or the resistance mechanisms among individual members of the *An. subpictus *complex to common insecticides in Sri Lanka or elsewhere. We therefore investigated a hypothesis that different sibling species of the *An. subpictus *complex vary in their susceptibility and resistance mechanisms to the common insecticides used for malaria vector control in Sri Lanka.

## Methods

### Mosquito collection and identification of sibling species of the Subpictus Complex

**Figure 1:**
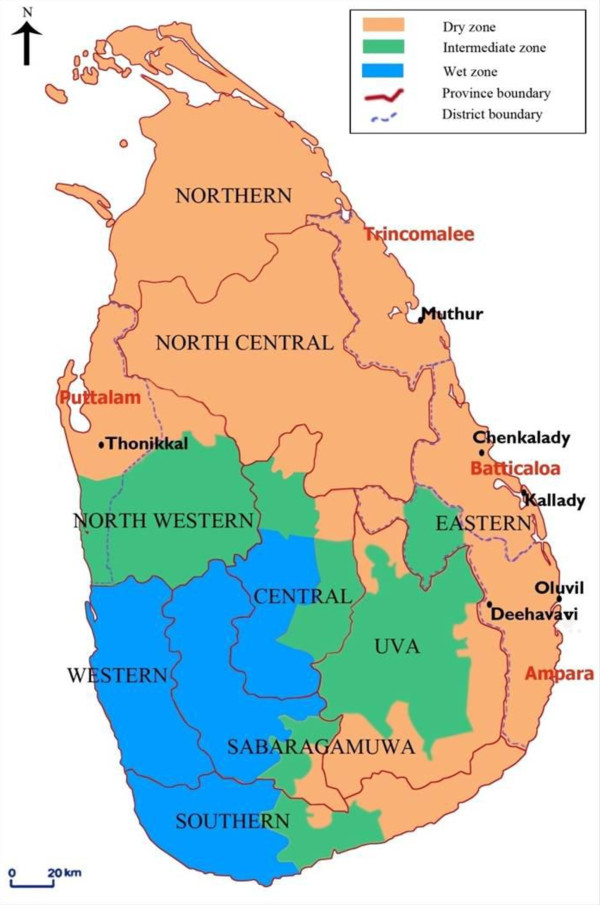
**Study sites in the dry zone of Sri Lanka**.

Adult female anopheline mosquitoes were collected monthly during the period between July 2008 and June 2010 at six sites *viz*. Oluvil (coastal locality) and Deehavavi (inland locality) in the Ampara district, Chenkalady (inland locality) and Kallady (coastal locality) in the Batticaloa district and Muthur (inland locality) in the Trincomalee district (inland locality) of the Eastern province and one inland site viz. Thonikkal in the Puttalam district of the Northwestern province of Sri Lanka (Figure 1). Inland localities are defined as being ≥ 2.5 km from the coast. Both cattle-baited hut (CBHC) and cattle-baited net (CBNC) collection techniques were used to collect blood-fed adult mosquitoes [22].

The collected adults were brought to the Zoology laboratory of the Eastern University and identified as *An. subpictus s.l*. using published keys [23]. Facilities and expertise were not available for determining polytene chromosome banding patterns during this investigation and therefore only morphological characteristics were used in identification. Identified blood-fed females were maintained individually and single female F_1 _progenies were raised as described previously [22]. Sibling species status of females laying eggs was determined through the reported number of ridges in the floats of egg that characterize the different sibling species in India [10]. Five to ten eggs from each female were placed on a clean microscopic slide and the number of ridges on floats was counted under a light microscope (x4, Olympus). Adults emerging from isofemale progenies identified as *An. subpictus *species B/*An. sundaicus s.l*., and *An. subpictus *species C and D were used for insecticide bioassays and biochemical assays to detect resistance status and underlying resistance mechanism to common insecticides. Due to the limited numbers of *An. subpictus *species A collected in most localities, they could only be tested in some of the insecticide bioassays.

### Insecticide bioassays

Insecticide-impregnated papers of DDT (4%), malathion (5%), deltamethrin (0.05%) and λ-cyhalothrin (0.05%) were prepared adopting standard World Health Organization (WHO) methods [24]. Whatman-No 1 filter papers in the size of 12 cm × 15 cm were used for impregnation with insecticides. Recommended discriminating dosages were prepared by mixing the technical grade insecticide with an appropriate spreading agent. Equal volumes of insecticide/oil solution (0.7 ml) and acetone (0.7 ml) were mixed and the resultant mixture was spread uniformly on the filter papers.

Insecticide bioassays were then carried out using the WHO standard bioassay kits [24]. Based on availability, 10-20 female progeny, aged 2-3 days from identified sibling species, were exposed to insecticide impregnated papers for one hour. At least four replicates for each insecticide were run in parallel. Papers impregnated with the carrier alone were used as controls. Dead mosquitoes were counted after a recovery period of 24 hours. If the control mortality was < 20% then the test mortality was adjusted using Abbott's formula [25]. A test mortality in the range between 98%-100%, 97%-80% and < 80% were respectively categorized as susceptible, possibly resistant (that requires verification) and resistant populations [26].

### Biochemical assays for insecticide resistance mechanisms

Biochemical assays on the enzymes concerned with insecticide resistance in mosquitoes were performed according to WHO procedures [27] essentially as described previously [19]. Adult female mosquitoes of isofemale progenies of identified sibling species were used in the assays. Individual mosquitoes of each identified sibling species were used for acetlcholinesterase (AChE), esterase, glutathione S-transferase (GST), monooxygenase and protein assays. Adult mosquitoes (n = 64) from each sibling species from every district were individually homogenised in 150 μl of ice cold distilled water. Fifty μl of the homogenate of individual mosquitoes was taken for the AChE assay and the remaining homogenate was centrifuged at 13, 000 g for two minutes and the supernatant used for the other assays.

### Acetylcholinesterase (AChE) assay

Two, 25 μl replicates of each mosquito homogenate were transferred to adjacent wells of a microtiter plate. The membrane bound AChE in the mosquito homogenate was solubilized by adding 145 μl of Triton phosphate buffer [1% (v/v) Triton X-100 in 0.1 M phosphate buffer pH 7.8] to each replicate aliquot. Ten μl of 0.01 M dithiobis 2-nitrobenzoic acid (DTNB) solution in 0.1 M M phosphate buffer pH 7.0 and 25 μl of the substrate 0.01 M acetylcholine iodide (AChI) were added to one replicate to initiate the reaction. To the other replicate 25 μl of AChI containing 10 μl of 0.1 M propoxur in acetone were added. The kinetics of the enzyme reaction was continuously monitored at 405 nm for 5 minutes in a microtiter plate reader (UV _max _Kinetic Plate Reader, Bio-Tek, USA). The percentage inhibition of AChE activity in the test well with propoxure compared to the uninhibited well was calculated. Residual activity > 70% suggests homozygosity for an altered AChE while values between 30% and 70% suggest heterozygosity [27].

### p-Nitrophenyl acetate (pNPA) esterase assay

Two hundred μl of 1 mM *p*-nitrophenyl acetate (*p*NPA) working solution (100 mM *p*NPA in acetonitrile: 50 mM sodium phosphate buffer pH 7.4, 1:100) were added to each 10 μl of each homogenate. The kinetics of the reaction was measured at 405 nm for 2 min at 22^0 ^C. An extinction co-efficient of 6.53 mM^-1 ^(corrected for a path length of 0.6 cm) was used to determine the concentration of the product. The *p*NPA esterase activity per individual mosquito was reported as μmol of product formed per min per mg protein.

### Glutathione-S-transferase (GST) assay

Ten μl of each homogenate was mixed with 200 μl of substrate solution (5 parts of 10.5 mM reduced glutathione in 100 mM phosphate buffer pH 6.5 + 5 parts of 63 mM 1-chloro 2,4 -dinitro-benzene in methanol) in a microtitre plate well. The reaction rate was measured at 340 nm for 5 min. An extinction coefficient of 5.76 mM^-1 ^(corrected for a path length of 0.6 cm) was used to determine the concentration of the product. Activities were reported as μmol of product formed per min per mg protein.

### Cytochrome p450 monooxygenase assay

Ten μl of homogenate was mixed with 80 μl of potassium phosphate buffer (pH 7.2) plus 200 μl of 6.3 mM tetramethyl benzidine (TMBZ) working solution (0.01 g TMBZ dissolved in 5 ml methanol and then in 15 ml of sodium acetate buffer pH5.0) plus 25 μl of 3% (v/v) H_2_O_2 _solution in a microtiter plate well. After 2 h incubation at room temperature, the plate was read at 630 nm as an end point assay. Values were compared with a standard curve of absorbance for known concentrations of cytochrome C. The values are expressed as equivalent units of cytochrome p450 per mg protein, correcting for the known haem content of cytochrome C and p450.

### Total protein

All mosquitoes assayed for enzyme activities were also analysed for their total proteins in order to calculate specific activities. Ten μl of each homogenate was mixed with 300 μl of Biorad protein assay working solution (prepared according to manufacturer's instructions) and was read at 630 nm. Protein values in mg ml^-1 ^were calculated for individual mosquitoes from a standard curve of absorbance of known concentrations of bovine serum albumin, according to manufacturer's instructions.

### Malathion metabolism assay

Batches (20-25) of adult mosquitoes were homogenized in 0.5 ml of 25 mM tris-HCl buffer (pH 7.5) and centrifuged at 13, 000 g for 5 minutes. The supernatant was incubated at room temperature with 300 μM malathion for 2 h. The samples were extracted with two volumes of 0.5 ml acidified chloroform and dried under an air current. The extract was resuspended in 30 μl acidified chloroform and loaded onto a silica gel thin layer chromatography plate. The plate was eluted with a mobile phase consisting of *n*-hexane: diethyl ether (1:3). After the run the plate was sprayed with a 0.5% (w/v) 2,6-dibromoquinone 4-chloromide in cyclohexane and left at 100°C for 2 hours to visualize spots of malathion and its metabolic products. Buffer (0.5 ml), incubated with 300 μM malathion and 300 μM sodium hydroxide (NaOH) was run as a positive control. Buffer (0.5 ml), incubated with the same concentration of malathion, served as negative control.

### Statistical analysis

The results of bio-assay experiments and the discriminating activity levels of different enzymes were analaysed using two-way ANOVA using Minitab statistical software (Minitab Inc, PA, USA). Chi-square tests were also done to compare the differential susceptibility of sibling species to each tested insecticide in each district using the same statistical software.

## Results

Data from the insecticide susceptibility bioassays are presented in Table 1. The two-way ANOVA revealed that there were significant variations in the susceptibility to the tested insecticides by sibling species but not districts. In comparison with *An. subpictus *species B/*An. sundaicus s.l*., *An. subpictus *sibling species C and D collected from all four districts were more resistant to all four insecticides, viz. DDT, malathion, deltamethrin and λ-cyhalothrin, that were tested. Where sufficient numbers of *An. subpictus *species A were available for testing, this species tended to be more resistant than *An. subpictus *species B/*An. sundaicus s.l*. except for deltamethrin in Trincomalee specimens. The levels of resistance in all four species were in the order DDT > malathion > pyrethroids.

**Table 1:** Susceptibility of *An.subpictus *sibling species populations to common insecticides (number of adults in each sibling species tested against each insecticide is ≥ 100)

Insecticide	District	Percentage mortality			
		
		A	B/*An. sundaicus s.l*.	C	D
DDT	Batticaloa	25 ± 2 (**R**)	38 ± 2 (**R**)	28 ± 1(**R**)	23 ± 4(**R**)
	
(5%)	Puttalam	ND	47 ± 1(**R**)	24 ± 2(**R**)	27 (**R**)
	
	Trincomalee	ND	41 ± 3 (**R**)	25 ± 2 (**R**)	26 ± 1 (**R**)
	
	Ampara	ND	41 ± 2 (**R**)	16 ± 3(**R**)	35 ± 3 (**R**)

Malathion	Batticaloa	ND	97 ± 2 (**V**)	62 ± 1 (**R**)	62 ± 1(**R**)
	
(4%)	Puttalam	ND	98 ± 2(**S**)	49 ± 4(**R**)	59 ± 1(**R**)
	
	Trincomalee	64 ± 1(**R**)	93 ± 4(**V**)	62 ± 1(**R**)	69 (**R**)
	
	Ampara	ND	97 ± 1(**V**)	63 ± 2(**R**)	61 ± 3(**R**)

Deltamethrin	Batticaloa	80 ± 3(**V**)	97 ± 1 (**V**)	82 ± 3(**V**)	93 ± 1 (**V**)
	
(0.05%)	Puttalam	ND	97 ± 3(**V**)	88 ± 2 (**V**)	96 ± 2(**V**)
	
	Trincomalee	100(**S**)	98 ± 1(**S**)	86 ± 2 (**V**)	94 ± 2(**V**)
	
	Ampara	ND	100(**S**)	88 ± 1 (**V**)	96 ± 2(**V**)

λ-Cyhalothrin	Batticaloa	ND	100(**S**)	85 ± 6 (**V**)	97 ± 1(**V**)
	
(0.05%)	Puttalam	ND	100(**S**)	72 ± 2 (**R**)	84 (**V**)
	
	Trincomalee	ND	100(**S**)	75 ± 2 (**R**)	88 ± 2(**V**)
	
	Ampara	ND	100(**S**)	77 ± 2(**R**)	86 ± 4 (**V**)

Mortality 98-100% indicates susceptibility (**S**), 97-80% suggests possibility of resistance that requires verification (**V**), < 80% indicates resistance (**R**) [26].

ND- not determined

Chi-square test revealed a significant difference (*p*< 0.05) between sibling species in their susceptibility to each tested insecticide in each district except for 4% DDT in the Batticaloa district. This difference is attributed to the greater susceptibility of *An. subpictus *species B/*An. sundaicus s.l*. to the tested insecticides, However significantly greater susceptibility was shown by species D compared to C for DDT 5% and deltamethrin 0.05% in the Ampara district and for λ-cyhalothrin in all districts except Ampara. Species A and C were also significantly more susceptible to deltamethrin 0.05% than species D in the Batticaloa district (*p *= 0.021) and Species A was more susceptible than species C and D to the same insecticide in the Trincomalee district (*p *< 0.001).

Based on previous published data on discriminating activity levels of the different enzymes in Sri Lanka, i.e. 0.25 μmol/mg/min esterase activity, 0.40 μmol/mg/min GST activity and 0.35 equivalent units of monooxygenase [19], the percentage of sibling species populations that showed more than these values are given in Table 2. Detailed data are presented as bar charts showing the esterase, glutathion-S-transferase and monooxygenase activities in Additional File 1 Additional file 2 and Additional file 3.

**Table 2:** Activities of insecticide detoxifying enzymes in *An.subpictus *sibling species in different districts

District	Species	^a^AChE (%)			^b^GST	^c^Est	^d^MO	^e^MCE
						
		< 30 [SS]	30-70 [RS]	> 70 [RR]				
Trincomalee	B/*An. sundaicus s.l*.	56	38	6	55	33	0	+
	
	C	25	40	35	49	42	0	++
	
	D	47	43	10	54	32	0	++

Batticaloa	B/*An. sundaicus s.l*.	53	44	3	27	36	0	+
	
	C	39	29	32	32	51	0	++
	
	D	40	34	26	23	42	0	++

Ampara	B/*An. sundaicus s.l*.	70	24	6	18	16	0	_
	
	C	47	25	28	53	58	0	++
	
	D	56	23	21	50	42	0	++

Puttalam	B/*An. sundaicus s.l*.	63	31	6	29	22	0	+
	
	C	44	36	20	40	58	0	++
	
	D	41	28	31	48	54	0	++

^a^Homozygous sensitive (**SS**), heterozygous (**RS**) and homozygous insensitive (**RR**) are given according to the percentage remaining activity of AChEs in individual mosquitoes after insecticide inhibition [27].

^b ^% population having glutathione S-transferase (GST) specific activity above 0.40 μmol/mg/min [19].

^c ^% population having esterase (Est) specific activity above 0.25 μmol/mg/min [19].

^d ^% population having monooxygenase (MO) levels above 0.35 per mg protein of cytochrome P_450_[19]

^e ^malathion carboxyesterase (MCE) activity as measured by malathion metabolism studies:**- **no activity, **+**- moderate activity (production of only either mono-acid or di-acid), **++**-high activity (production of both mono-acid and di-acid).

Detailed results of the assays conducted to identify the insensitivity of AChE to insecticide inhibition by propoxur are presented in Additional File 4. Populations with more than 70% remaining activity after inhibition can be categorized as homozygous resistance (RR) with respect to altered AChE mechanism. Populations with 30-70% and less than 30% remaining activity can be categorized as heterozygous (RS) and homozygous susceptible (SS) respectively [27]. Percentage of different sibling species populations having RR, RS and SS individuals for insensitive AChE mechanisms are shown in Table 2. Statistical analysis for RR and RS populations of each sibling species showed significant variation among the three species (F = 12.14; DF = 2; P = 0.008) without any significant variation between sites (F = 4.71; DF = 3; P = 0.051). This was attributed to greater susceptibility of *An. subpictus *B/*An. sundaicus s.l*. AChE to propoxur inhibition. Similarly variation in esterase activity between the species was also found to be significant (F = 7.67; DF = 2; P = 0.022, with *An. subpictus *B/*An. sundaicus s.l*. having a lower activity than *An. subpictus *species C and D), but not significantly dependent on location (F = 4.88; DF = 3; P = 0.238). Since none of the populations were found to have monooxygenase activity beyond the discriminative activity values statistical analysis was not performed for monooxygenase activity.

Although GST activity between the sibling species was found to be significantly different, with that for *An. subpictus *B/*An. sundaicus s.l*. being lower except in Trincomalee (F = 5.43; DF = 2; P = 0.044), the activity did not vary significantly with the district where the specimens were collected (F = 3.14; DF = 3; P = 0.108).

Sibling species C and D tended to metabolise malathion into mono- and di-acid products at a faster rate than the *An. subpictus *B/*An. sundaicus s.l*. with a higher activity of malathion carboxylesterase (Table 2).

## Discussion

Our findings show that the different sibling species of the *An. subpictus *complex vary in their susceptibility and resistance mechanisms to the common insecticides used by the AMC for vector control in Sri Lanka. Resistance to insecticides in insects can be due to changes in the activity of insect enzyme systems that leads to detoxification of insecticides, sequestration of insecticides away from target molecules or alterations in the insecticide target site that lower binding or effector function of the insecticide. Increased metabolic activities are associated with monooxygenases, GSTs or esterases [17,18]. Esterases can offer resistance to organophosphates (e.g. malathion), carbamates (e.g. propoxur) and pyrethroids (e.g. deltamethrin). GSTs can confer resistance to organophosphates, organochlorines (e.g. DDT) and pyrethroids. Mutations in the target site of AChE and insensitivity of sodium channels protect insects from organophosphates, and pyrethroids and DDT respectively. Insects are also protected from the toxic effect of pyrethroids by monooxygenases [17,18].

Sri Lanka has gone through different insecticide regimes at different times over the last six decades for malaria control [15,19,20]. In recent times deltamethrin has been used in the districts of Ampara, Trincomalee and Puttalam whereas deltamethrin, cyfluthrin and λ-cyhalothrin have been used for IRS in the Batticaloa district [15]. IRS with DDT was first used throughout the island from 1947 to1955. After a period of discontinuation, DDT was re-introduced in vector control programme in 1958. DDT resistance in major malaria vector *An. culicifacies *was first reported in 1969 [28]. As a result of DDT resistance in the major malaria vectors and environmental and health considerations, DDT was replaced by the organophosphate malathion in the period 1975 to 1977. Vector resistance to DDT declined slowly after cessation of its usage, but increased again after 1983 due to a GST-based resistance mechanism, which was first postulated to be selected by exposure to DDT and subsequently through exposure to organophosphates [21,29]. GSTs act on DDT and convert it into dichloro diphenyl ethylene (DDE) and hence the GSTs are referred to as DDT dehydrochlorinases (DDTase) [17]. Our findings now show that all the *An. subpictus *populations tested are resistant to DDT and have high DDT dehydrochlorinase activity, which is probably responsible for the DDT resistance in these populations. Although, *An. subpictus *B/*An. sundaicus s.l*. is moderately resistant to DDT, the relatively lower GST activity in this species in different localities is consistent with the possible involvement of a different mechanism of resistance. Mutation of the voltage-gated sodium ion channel proteins, the target of DDT and pyrethroids, causing DDT resistance has been previously reported in Sri Lankan *An. subpictus s.l*. [20].

Organophosphates and carbamates inhibit the enzyme acetyl cholinesterase AChE. Higher remaining activity of AChEs when inhibited with the standard dosage of the carbamate propoxur shows the insensitivity of the target site that is associated with resistance to organophosphates and carbamates [17,18]. Relative susceptibility to malathion of *An. subpictus *B /*An. sundaicus s.l*. populations, observed in the insecticide bioassay is consistent with a lower proportion of RR or resistant forms of AChE in the AChE assay. A previous study carried out using *An. subpictus *collected from five different districts in Sri Lanka showed that the highest level of insensitivity to AChE were in *An. subpictus s.l*. collected from an inland locality of Trincomalee with a high homozygous insensitive population according to WHO classification [19]. This can be caused by a dominance of the sibling species C and D in the inland areas of Trincomalee [11].

The lower malathion carboxylesterase and esterase activities observed in enzyme assays provide an explanation for the greater malathion susceptibility of *An. subpictus *B /*An. sundaicus s.l*. compared to *An. subpictus *species C and D in the insecticide bioassay. However moderate levels of resistance to malathion are shown by species C and D. High enzyme activity level of esterase and GSTs and the presence of altered target site AChEs in these populations might have contributed to this. GSTs are important in organophosphate resistance. Since high GST levels were further selected by the organophosphates, that were introduced after the cessation of DDT use in 1975/77, the vector populations are still resistant to DDT in the absence of DDT use for nearly three and a half decades as also earlier reported by Herath et al. [21]. Metabolic resistance to organophosphates in insects can be due to mutated enzymes that metabolize the insecticide more rapidly [17,18]. The results of malathion metabolism study supports the previous report [30] that carboxylesterase mediated resistance is the major mechanism against malathion in *An. subpictus *species C and D. This may be the result of the widespread use of malathion for IRS from mid 1970s to early 1990s. In nearby India, *An. subpictus s.l*. populations showed variable resistance to DDT and malathion [7]. Similar to India, malathion had only been permitted for use in malaria control in Sri Lanka [7,30], and therefore the observed malathion resistance might have been selected as a direct consequence of malaria control activities [30].

Mutated sodium ion channel proteins and elevated monooxygenases are found in pyrethroid resistance populations of malaria vectors in Sri Lanka [20]. Absence of elevated activity of monooxygenases in all populations from four districts indicates the little involvement of these enzymes in metabolic resistance in the *An. subpictus *B/*An. sundaicus s.l., An. subpictus *species C and D populations tested in the present study. Monooxygenases are important in providing pyrethroid resistance [18]. Pyrethroids were introduced in the country in early 1990s and initially used in selected endemic localities. Our results show that the populations of *An. subpictus *B/*An. sundaicus s.l*. tested are all highly susceptible to pyrethroids. However a differential susceptibility to different pyrethroids, as observed here for deltamethrin and λ-cyhalothrin, has also been reported previously for vector mosquitoes [19,31]. This differential susceptibility may be due to the different chemical structures causing differential metabolism and access to target molecules, as well as the nature of possible mutations conferring resistance in the target sodium channel [reviewed in [17]. Tests on the sensitivity of sodium ion channel regulatory proteins could not be performed in this study due to resource limitations. Both elevated esterases and insensitivity of the sodium ion channel may contribute to the low levels of pyrethroid resistance seen among the populations of *An. subpictus *species C and D.

*An. subpictus *B/*An sundaicus s.l*. is more prevalent in coastal areas while *An. subpictus *sibling species C and D are found in greater numbers in inland areas of Sri Lanka [11,14]. IRS in Sri Lanka is carried out mainly in inland areas and thus inland populations are under greater selection pressure. A previous study in the North-central province of Sri Lanka revealed a significance association in feeding and resting behavior among sibling species and showed that species B prefers to feed and rest outdoors and species A and C in indoors [reviewed in [2]. This may partly explain why *An. subpictus *species C and D are more resistance to common insecticides than the more coastal *An. subpictus *B/*An sundaicus s.l*. Our findings are also consistent with the possibility that the majority if not all of the mosquitoes identified as *An. subpictus *B/*An. sundaicus s.l*. are in fact *An. sundaicus s.l*. and that *An. sundaicus s.l*. is genetically more susceptible to the tested insecticides [32,33]. Control of *An. sundaicus s.l*. in coastal Southeast Asia has recently relied more on environmental management strategies such as elimination of brackish water breeding sites than on the use of insecticides [32].

The incidence of malaria has recently fallen sharply in Sri Lanka, and this can largely be attributed to the use of insecticides to control vector populations [2,15]. The success of a malaria control or eradication program in the island and many other countries therefore relies heavily on monitoring genetically different vector populations and their sensitivity to varying insecticides. Our findings indicate an important need in this context for developing a simple molecular tool to differentiate *An. subpictus *sibling species from each other and from *An. sundaicus s.l*. in field studies.

## Conclusions

The differences in insecticide susceptibility and insecticide resistance mechanism shown by members of the Subpictus Complex are important considerations for developing the malaria control and eradication program in Sri Lanka. Similar studies on species complexes of other anopheline vectors of malaria are necessary for effective malaria control worldwide. The findings are also consistent with most if not all morphologically identified *An. subpictus *species B in Sri Lanka belonging to the Sundaicus Complex. There is a need therefore to develop molecular techniques that can be used to differentiate morphologically similar anopheline species in field situations for more effective vector control.

## Competing interests

The authors declare that they have no competing interests.

## Authors' contributions

SNS, SHPPK and RR conceived the study. PJJ performed all the field collections. PJJ and TCW did laboratory studies. PJJ, SNS and SHPPK did analysis. SNS, SHPPK and RR wrote the manuscript. All authors read and approved the final manuscript.

## Supplementary Material

Additional file 1 **Distribution of esterase enzyme activities in *An. subpictus *sibling species B/*An. sundaicus s.l., An. subpictus *species C and D collected from four districts (A-Trincomalee, B- Ampara, C- Puttalam, D- Batticaloa) of Sri Lanka**. Click here for file

Additional file 2 **Distribution of glutathione-S-transferase enzyme activities in *An. subpictus *sibling species B/*An. sundaicus s.l., An. subpictus *species C and D collected from four districts (A-Trincomalee, B- Ampara, C- Puttalam, D- Batticaloa) of Sri Lanka**. Click here for file

Additional file 3 **Distribution of monooxygenase enzyme activities in *An. subpictus *sibling species B/*An. sundaicus s.l., An. subpictus *species C and D collected from four districts (A-Trincomalee, B- Ampara, C- Puttalam, D- Batticaloa) of Sri Lanka**. Click here for file

Additional file 4 **Distribution acetylcholinesterase activity after inhibition with propoxur as a proportion of activity without inhibition in *An. subpictus *sibling species B/*An. sundaicus s.l., An. subpictus *species C and D collected from four districts (A-Trincomalee, B- Ampara, C- Puttalam, D- Batticaloa) of Sri Lanka**. Click here for file
