# Supplementary figures and images for: Variations in susceptibility to common insecticides and resistance mechanisms among morphologically identified sibling species of the malaria vector Anopheles subpictus in Sri Lanka
Source: Parasit Vectors. 2012 Feb 10;5:34. doi: 10.1186/1756-3305-5-34 (PMC3317438; doi:10.1186/1756-3305-5-34)

## Slide 1
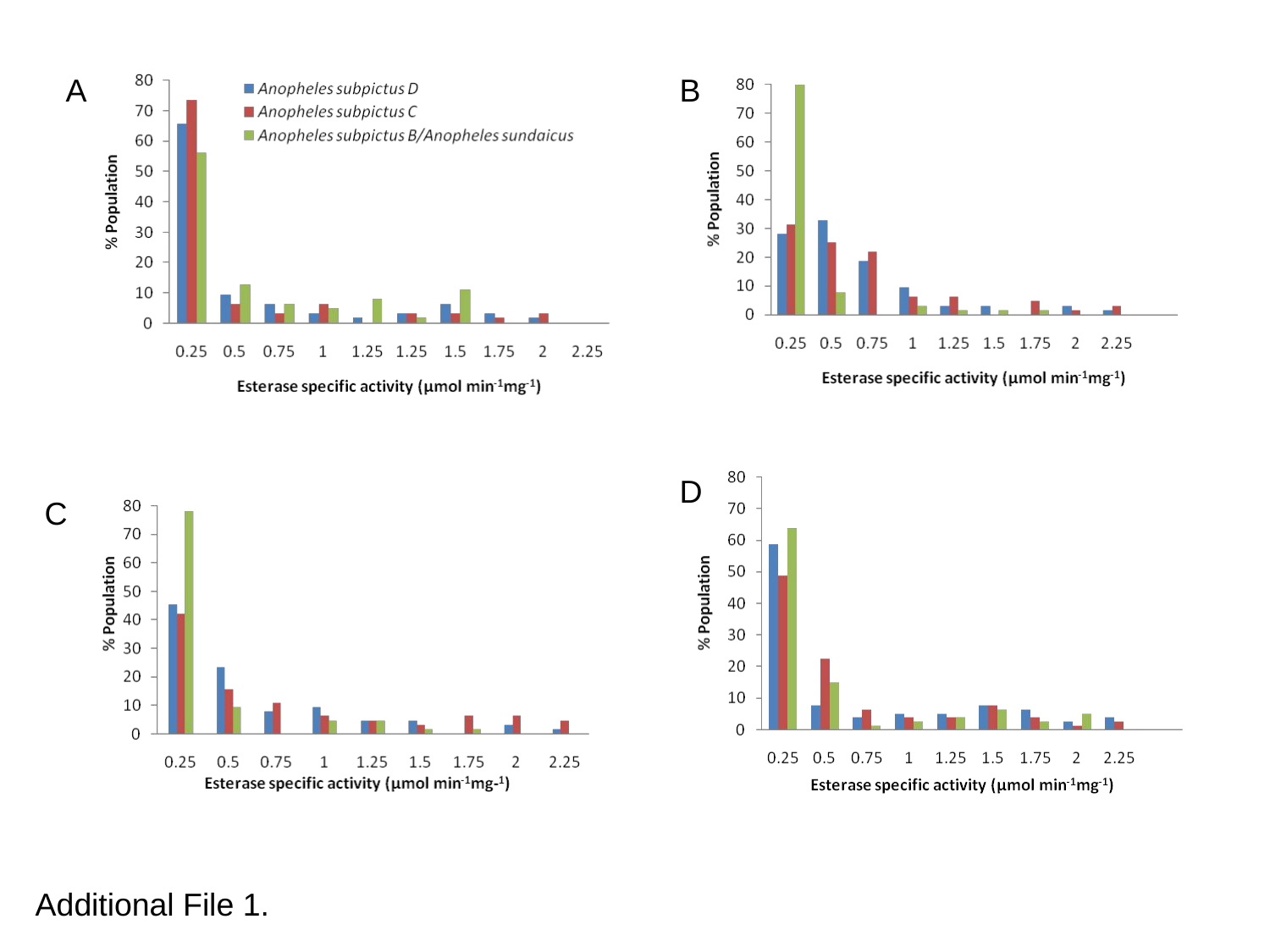

A
B
D
C
Additional File 1.

Supplement: Additional file 1 — Distribution of esterase enzyme activities in An. subpictus sibling species B/An. sundaicus s.l., An. subpictus species C and D collected from four districts (A-Trincomalee, B- Ampara, C- Puttalam, D- Batticaloa) of Sri Lanka. [file 1756-3305-5-34-S1.PPT]

## Slide 1
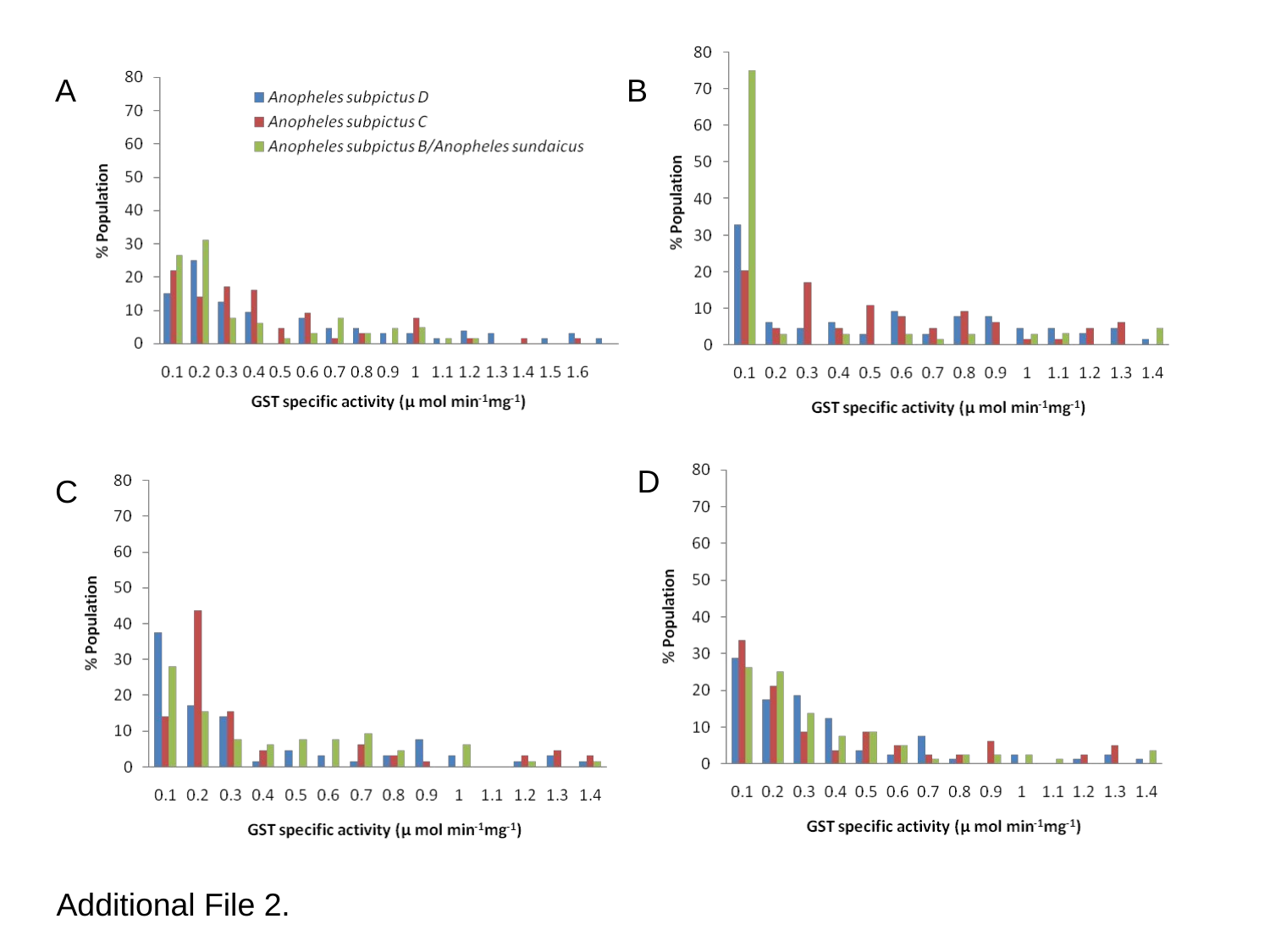

A
B
D
C
Additional File 2.

Supplement: Additional file 2 — Distribution of glutathione-S-transferase enzyme activities in An. subpictus sibling species B/An. sundaicus s.l., An. subpictus species C and D collected from four districts (A-Trincomalee, B- Ampara, C- Puttalam, D- Batticaloa) of Sri Lanka. [file 1756-3305-5-34-S2.PPT]

## Slide 1
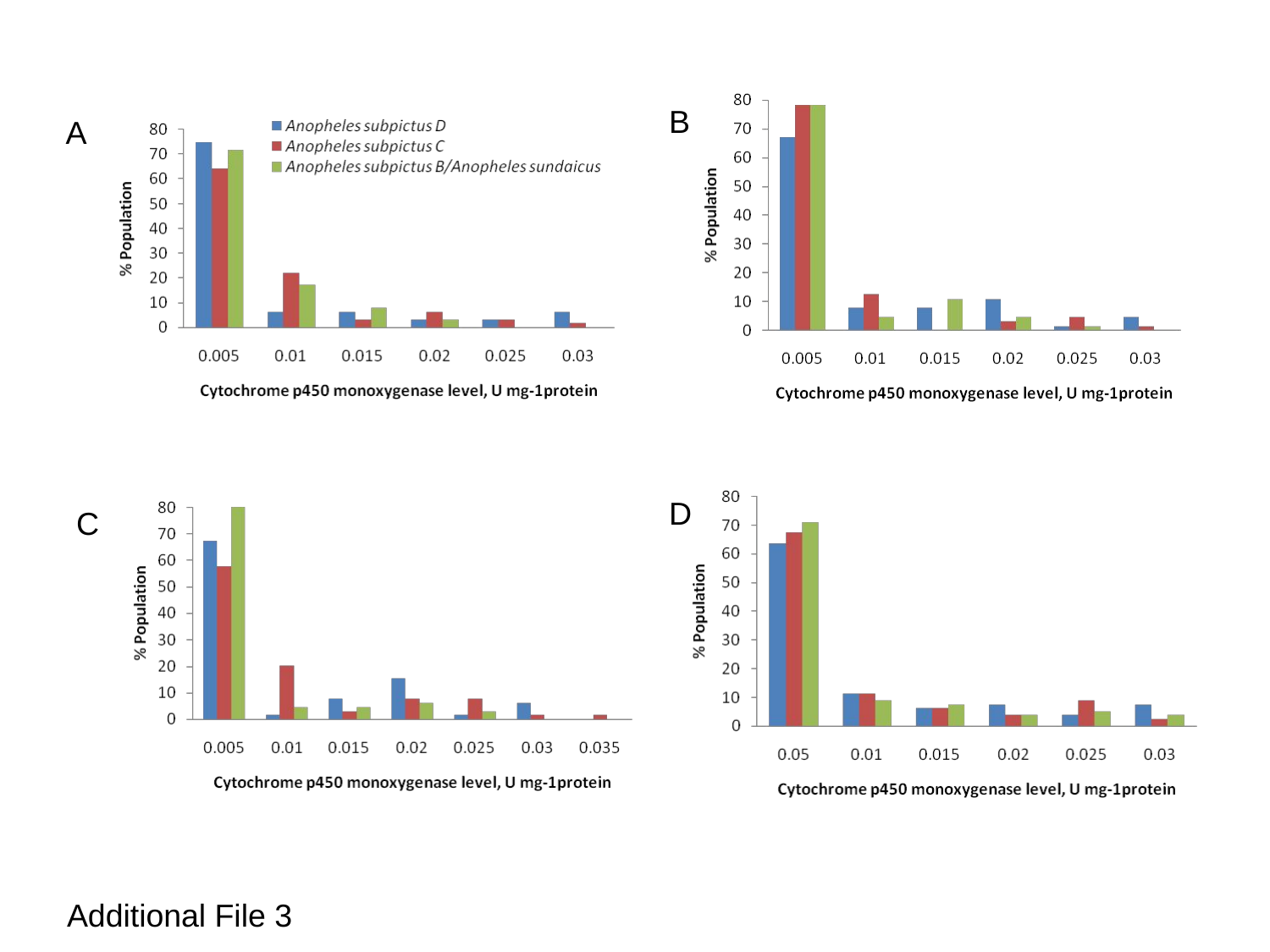

B
A
D
C
Additional File 3

Supplement: Additional file 3 — Distribution of monooxygenase enzyme activities in An. subpictus sibling species B/An. sundaicus s.l., An. subpictus species C and D collected from four districts (A-Trincomalee, B- Ampara, C- Puttalam, D- Batticaloa) of Sri Lanka. [file 1756-3305-5-34-S3.PPT]

## Slide 1
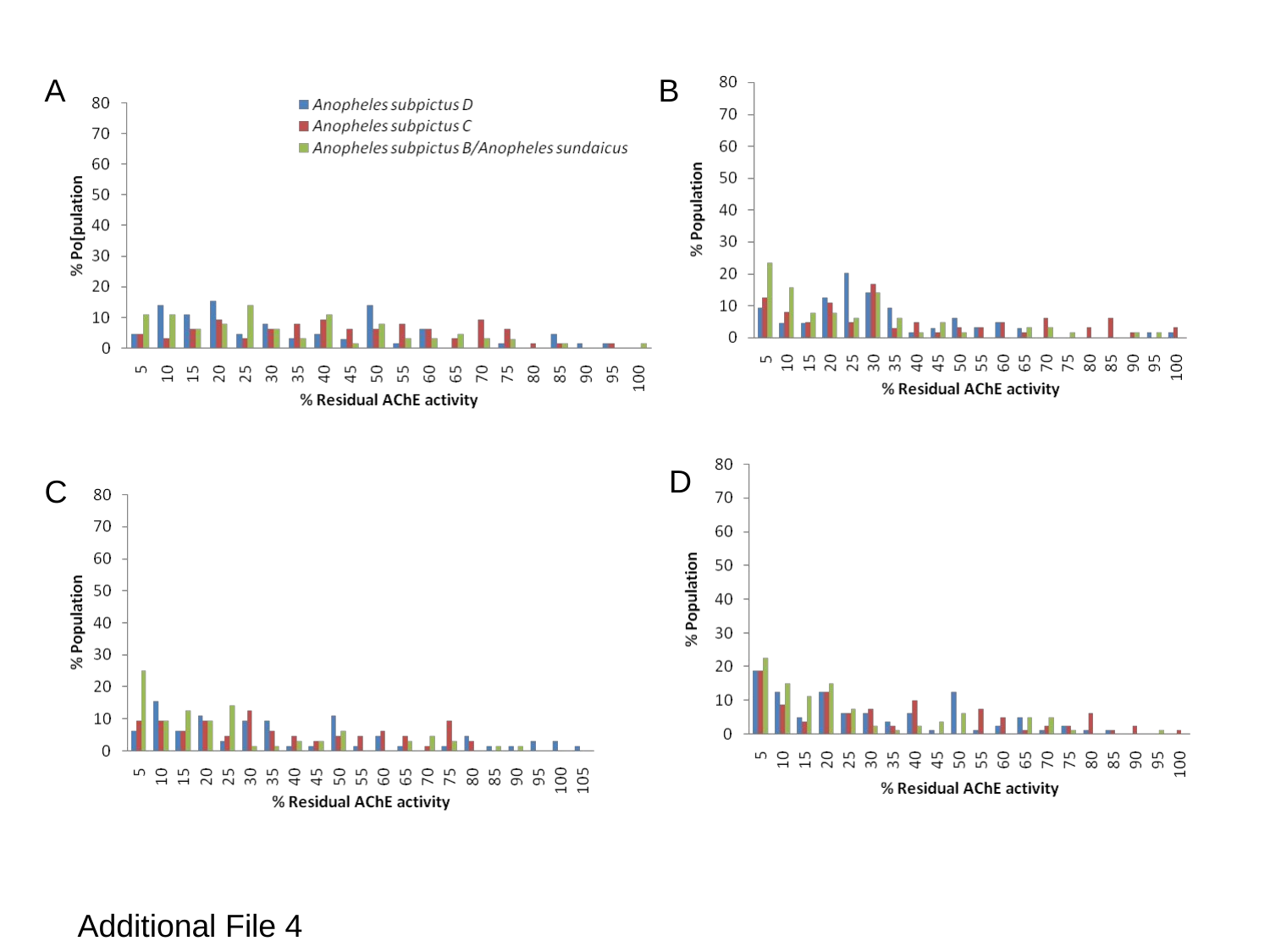

A
B
D
C
Additional File 4

Supplement: Additional file 4 — Distribution acetylcholinesterase activity after inhibition with propoxur as a proportion of activity without inhibition in An. subpictus sibling species B/An. sundaicus s.l., An. subpictus species C and D collected from four districts (A-Trincomalee, B- Ampara, C- Puttalam, D- Batticaloa) of Sri Lanka. [file 1756-3305-5-34-S4.PPT]
